# The advanced care coordination program: a protocol for improving transitions of care for dual-use veterans from community emergency departments back to the Veterans Health Administration (VA) primary care

**DOI:** 10.1186/s12913-019-4582-3

**Published:** 2019-10-22

**Authors:** Lindsay B. Miller, Heidi Sjoberg, Ashlea Mayberry, Marina S. McCreight, Roman A. Ayele, Catherine Battaglia

**Affiliations:** 1 Department of Veterans Affairs, Eastern Colorado Health Care System, 1700 N. Wheeling St, Aurora, CO 80045 USA; 2 University of Colorado, Anschutz Medical Campus, Colorado School of Public Health, 13001 E. 17th Pl, Aurora, CO 80045 USA

**Keywords:** Care coordination, Veterans, VA, Veterans health administration, Emergency department, Social determinants of health, Longitudinal, Social work, Care transitions

## Abstract

**Background:**

Veterans who access both the Veterans Health Administration (VA) and non-VA health care systems require effective care coordination to avoid adverse health care outcomes. These dual-use Veterans have diverse and complex needs. Gaps in transitions of care between VA and non-VA systems are common. The Advanced Care Coordination (ACC) quality improvement program aims to address these gaps by implementing a comprehensive longitudinal care coordination intervention with a focus on Veterans’ social determinants of health (SDOH) to facilitate Veterans’ transitions of care back to the Eastern Colorado Health Care System (ECHCS) for follow-up care.

**Methods:**

The ACC program is an ongoing quality improvement study that will enroll dual-use Veterans after discharge from non-VA emergency department (EDs), and will provide Veterans with social worker-led longitudinal care coordination addressing SDOH and providing linkage to resources. The ACC social worker will complete biopsychosocial assessments to identify Veteran needs, conduct regular in-person and phone visits, and connect Veterans back to their VA care teams.

We will identify non-VA EDs in the Denver, Colorado metro area that will provide the most effective partnership based on location and Veteran need. Veterans will be enrolled into the ACC program when they visit one of our selected non-VA EDs without being hospitalized. We will develop a program database to allow for continuous evaluation. Continuing education and outreach including the development of a resource guide, Veteran Care Cards, and program newsletters will generate program buy-in and bridge communication. We will evaluate our program using the Reach, Effectiveness, Adoption, Implementation, and Maintenance framework, supported by the Practical, Robust Implementation and Sustainability Model, Theoretical Domains Framework, and process mapping.

**Discussion:**

The ACC program will improve care coordination for dual-use Veterans by implementing social-work led longitudinal care coordination addressing Veterans’ SDOH. This intervention will provide an essential service for effective care coordination.

## Background

Veterans with complex health care needs increasingly utilize both the Veterans Health Administration (VA) and non-VA hospitals [1–3]. Roughly 50% of Veterans are dual-users [2–4], receiving care from both VA and non-VA hospitals. With the addition of the VA’s Mission Act (previously the Choice Act), access to community care will likely increase. Allowing Veterans to receive community care improves their access to health care [3, 5]; however, coordination between VA and non-VA hospitals is often a complex, multi-level, fragmented process [6, 7]. Effective care coordination for this dual-use population is essential to avoid adverse health care outcomes [8–10].

Currently, gaps are common in systematic methods of care coordination between the VA and non-VA hospitals. As a result, some Veterans must manage their own care coordination [11] or rely on VA primary care clinics to facilitate care coordination with non-VA hospitals, which causes fragmented care for this vulnerable population [12]. Among Veterans who have diverse and complex needs, including psychosocial stressors and functional limitations, self-managed care coordination is even less reliable [13, 14]. Within the VA, the multidisciplinary Patient Aligned Care Teams (PACTs) consisting of primary care providers, social workers, nurses, and administrative clerks, are often not notified of Veterans’ non-VA hospitalizations or emergency department (ED) visits, and are therefore limited in their ability to assist with care coordination outside of the VA. Without standardized care, dual-use Veterans are at a higher risk of adverse outcomes [9, 10] including an increased probability of readmission to the hospital within 30 days [9, 15], ED visits and hospitalizations [9, 16], conflicting treatments and duplicated tests [17–19], medication errors [20–23], and decreased satisfaction with their care [24].

Successful care coordination programs are imperative to assist the VA in meeting its goal of providing high-quality, timely access to care for Veterans [24, 25], and to reduce the possibility of adverse outcomes for dual-users [8–10]. Veterans are most in need of effective care coordination during vulnerable periods of transition [8, 26] (i.e. transitioning from an ED back to their home). During this transition, our program will provide Veterans with longitudinal care coordination that addresses their social determinants of health (SDOH). SDOH are the biopsychosocial circumstances of someone’s life, including neighborhoods where people live, health and access to quality health care, social and community contexts, access to education and economic stability [27]. Our program will assist with vulnerabilities in Veterans’ SDOH and will link them to essential resources.

This social-work led program will complete comprehensive assessments to evaluate each Veteran’s SDOH and their areas of need. Complete care coordination will include health assessments that review medical, psychosocial, behavioral health, and functional needs, regular phone contact and home and community visits as needed, and utilization of a multidisciplinary approach. This program will add value to the pre-existing care coordination system by efficiently using VA and community resources and by avoiding the duplication of health care services provided to Veterans, as duplicated services could create unnecessary confusion. Additionally, as the Eastern Colorado Health Care System (ECHCS) and non-VA hospitals are equal participants in this collaborative program, internal and external stakeholder engagement is paramount to the success of an enhanced care coordination program.

### Goals & Objectives

The Advanced Care Coordination (ACC) program is designed to be an ongoing quality improvement (QI), social worker-driven program that provides longitudinal care coordination for up to 90 days to address SDOH for dual-use Veterans who access non-VA EDs. The objectives of the ACC program are 1) to identify gaps in the collaboration process between the ECHCS and non-VA health care systems and to decrease non-VA ED use due to SDOH, and 2) to implement a comprehensive longitudinal care coordination intervention with a focus on Veterans’ SDOH that will enhance existing care coordination within the ECHCS and improve continuity of care for Veterans. With the Mission Act in place, timely care coordination is required. This care coordination program aims to reduce adverse outcomes for dual-users by enhancing care coordination services and improving access to high quality, timely, efficient care for dual-use Veterans.

The ACC program was modeled after our existing Veteran-centered, nurse-led care coordination programs, the Community Hospital Transitions Program (CHTP) and the rural Transitions Nurse Program (TNP). The CHTP was designed to provide short-term care coordination to Veterans who use both VA and non-VA health services and have been hospitalized at a non-VA facility [28]. The TNP facilitates care transitions for Veterans who are discharged from a VA hospital back to their rural VA primary care teams [29]. Both the CHTP and rural TNP have been disseminated successfully to other Veterans Affairs Medical Centers (VAMCs).

## Methods/design

### Study setting

Drawing on insights from key informant interviews completed for the implementation of CHTP, the ACC program will address identified gaps in care coordination by implementing social worker-led longitudinal care coordination between the VA and non-VA EDs. The ACC program will initially be implemented through the ECHCS Rocky Mountain Regional VAMC (RMR VAMC) in Aurora, Colorado. We plan to expand this program to the VA Nebraska-Western Iowa Health Care System after the program has been implemented at the ECHCS. Lessons learned during the development of the CHTP will be utilized to inform a successful implementation of the ACC program.

### Participant recruitment

The ACC program will provide comprehensive care coordination for dual-use Veterans who access non-VA EDs within the Denver, Colorado metro area. We will identify non-VA EDs in the areas that experience a large volume of Veteran admissions based on data available through the VA Business Office. From these data, we will determine which non-VA EDs will provide the most effective partnership based on location in relation to the RMR VAMC and Veteran need. We anticipate that our enrolled sample size will be 250–300 Veterans per year. Staff from our selected non-VA EDs will be asked by the ACC social worker to call us or to fax the Veteran’s History and Physical to notify us that a Veteran has been admitted to their non-VA ED. If the Veteran was discharged home from the non-VA ED, the ACC social worker will reach out to and enroll the Veteran into the ACC program. Veterans who are hospitalized in non-VA hospitals will not be enrolled into the ACC program and will be referred to the CHTP to avoid duplication of services.

Veterans who access our selected non-VA EDs will be eligible for enrollment into the ACC program if they meet the following criteria: 1) the Veteran is already receiving care through the ECHCS VA, or 2) the Veteran would like to establish care at the ECHCS VA. Veterans who are already enrolled into services in the ECHCS and are receiving ongoing case management from an ECHCS social worker will not be eligible for the ACC program as this would be a duplication of services. Additionally, Veterans who are discharged from the non-VA ED to a skilled nursing facility, long-term care facility, or assisted living facility will be excluded from the ACC program as they will receive case management at these respective facilities. Veterans with no working phone or who have been unable to reach after being called three times by the ACC social worker over a period of 2 weeks will also be excluded. Veterans who do not want to receive care through the VA will not be enrolled into the ACC program.

### Intervention description

**Fig. 1:**
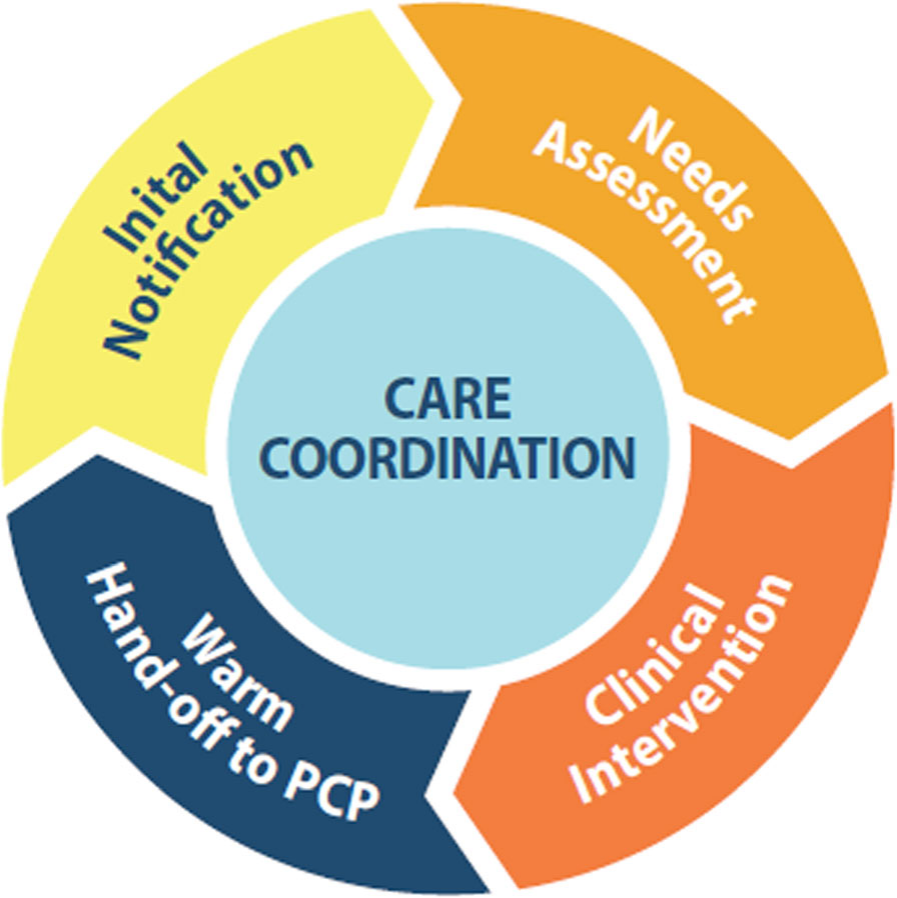
The Advanced Care Coordination Program Core Components

The ACC program consists of four core components (Fig. 1 The Advanced Care Coordination Program Core Components): 1) notification from the non-VA ED of a Veteran’s visit, 2) comprehensive needs assessment addressing SDOH including access to health care, economic status, housing status, psychological status, and social support, 3) individualized clinical interventions consisting of phone calls and home/community visits to link the Veteran to necessary resources in the VA and community, and 4) transfer of care back to the Veteran’s assigned VA primary care team.

Upon notification of a Veteran’s non-VA ED visit, the ACC social worker will review the Veteran’s VA medical chart to assess for program eligibility. The ACC social worker will collaborate with non-VA case managers, social workers, and discharge planners to stay informed of the Veteran’s non-VA ED admissions and discharges, and will provide ECHCS PACTs, ECHCS specialty clinics, and other appropriate providers with information about the Veteran’s non-VA ED visits for future coordination of care.

If the Veteran is eligible for enrollment, the ACC social worker will call the Veteran the following business day post non-VA ED discharge to enroll the Veteran into the ACC program. During this call the ACC social worker will complete the Social Workers Comprehensive Assessment, a validated tool in the VA’s health record system, to determine necessary clinical interventions. This needs assessment, mentioned in core component 2, is utilized by social workers throughout the national VA system to assess Veterans’ SDOH. During the assessment the ACC social worker will evaluate the Veteran’s biopsychosocial and clinical circumstances and will then utilize clinical judgment to determine the Veteran’s acuity level based upon the Veteran’s number and severity of SDOH needs. This assessment will establish the Veteran’s acuity level on a ranking from 1 (having minimal needs) to 4 (having significant needs), which the ACC social worker will use to guide further interventions.

Based upon the Veteran’s acuity level, the ACC social worker will intervene with the goal of improving the Veteran’s health outcomes and reducing the Veteran’s total health care costs using the VA Office of Community Care (VA OCC) Care Coordination guidelines as a resource. The VA OCC guidelines state that the VA must provide seamless Veteran-centered care coordination and collaboration between the VA and non-VA providers, in order provide Veterans with quality and timely health care services. Additionally, the VA OCC website outlines how best to coordinate quality health care services for Veterans with the VA [30]. The ACC program adheres to these guidelines by enhancing this seamless transition through coordinating care for Veterans post non-VA ED discharge.

The ACC social worker will provide varying degrees of case management to each Veteran. Veterans with a lower acuity will be enrolled into the ACC program for 2 weeks or less. Veterans with a higher acuity level will be enrolled into the ACC program for up to 90 days post non-VA ED discharge. The 90-day limit is based on a demonstrated gap in Veteran care during transition periods in the first 90 days post non-VA ED discharge. As part of the clinical intervention, the ACC social worker will provide weekly or bi-weekly phone calls, and if deemed necessary will visit the Veteran in his/her home or in the community if the Veteran is homeless, to complete clinical interventions. These clinical interventions will address needs regarding the Veteran’s SDOH. The interventions will help link the Veteran to resources (both non-VA and VA), such as transportation, rent resources, housing resources, food assistance, financial assistance, home health care, mental health and primary care, and substance use treatment. The ACC social worker will utilize teach-back methodology and Motivational Interviewing techniques to understand and address gaps in discharge preparedness and disease care management. Finally, the ACC social worker will complete a warm hand-off of to the ECHCS PACTs once the clinical interventions have been completed. If the Veteran is still in need of case management services after 90 days of assistance through the ACC program, the ACC social worker will connect the Veteran with a PACT social worker who works closely with the Veteran’s assigned primary care physician for continued case management. Data collected from these interventions will be stored in the ACC program database.

### Education

We will provide frequent outreach and continuing education to both the ECHCS and non-VA providers to elicit program feedback and ensure effective collaboration between the ACC social worker, the non-VA ED partners, the ECHCS PACTs, and Veterans admitted to non-VA EDs. Before implementing the program, we will conduct initial in-service meetings with each partner non-VA ED and related VA departments to discuss the program’s benefits and responsibilities. These meetings will promote awareness and collaboration during the program’s initial rollout. After the program’s initiation, we will provide frequent in-services to non-VA ED staff as well as ECHCS providers to facilitate information transfer with the ECHCS and to educate them on program adaptations as well as to elicit feedback for program improvement. During these in-services we will also solicit program feedback from the meeting participants to enhance collaboration and adapt the ACC program as necessary. We will design a transitions of care resource guide modeled on the resource guide employed by the CHTP [28], which we will disseminate to non-VA partners as a method of providing information on internal ECHCS resources and program components. In addition, a one-page fact sheet will be disseminated to non-VA and ECHCS partners informing them of the program and essential ECHCS resources to coordinate Veteran care.

Our education in non-VA EDs will be supplemented by Veteran outreach, which we will accomplish by creating a Veteran Care Card that we will mail out to Veterans who participate in the ACC program. The Care Card will include relevant care coordination resources including direct secure phone and fax numbers for the ACC program, resources for the VA Billing department, and the name of the Veteran’s ECHCS Primary Care Provider. We will also send out a letter with the Care Card that further outlines care coordination resources for the Veterans and that includes their PACT social workers’ contact information. Once Veterans have completed their involvement with the ACC program we will mail them a graduation letter with pertinent information regarding resources for the Veterans, goals that were worked on during their involvement with the ACC program, goals the Veterans are continuing to work on, and pertinent provider contact information. With the essential information at hand, these Veterans will have a one-stop-resource to initiate care coordination when admitted to non-VA EDs.

### Partnership approach

#### Operational partners

This project will be carried out in partnership with the VA OCC, ECHCS stakeholders, and the Veterans Integrated Service Network (VISN) 19. The ACC program is funded through the Office of Veterans Access to Care (OVAC) and the Office of Rural Health (ORH). These departments provide protected time for project personnel to participate in this QI intervention. The ACC program has been integrated into the Triple-Aim Quality Enhancement Research Initiative (QUERI) Program, which funded the CHTP. QUERI has funded these programs with the purpose of supporting QI interventions to enhance Veteran care and promoting learning healthcare systems as well as enhancing clinical practice through research. The proposed program will enhance ECHCS and non-VA partnerships by facilitating care coordination among non-VA ED utilizers.

#### Evaluation team

We have a multidisciplinary team consisting of experts in qualitative and quantitative research, statistics, data management, implementation science, clinical intervention specialists and consultants, public health, nursing, medicine, health economics, social work, and a national training educator. Our team has unique and diverse expertise that allows us to evaluate the implementation of a comprehensive care coordination program in the VA and its expansion to other sites.

#### Evaluation of intervention

**Fig. 2:**
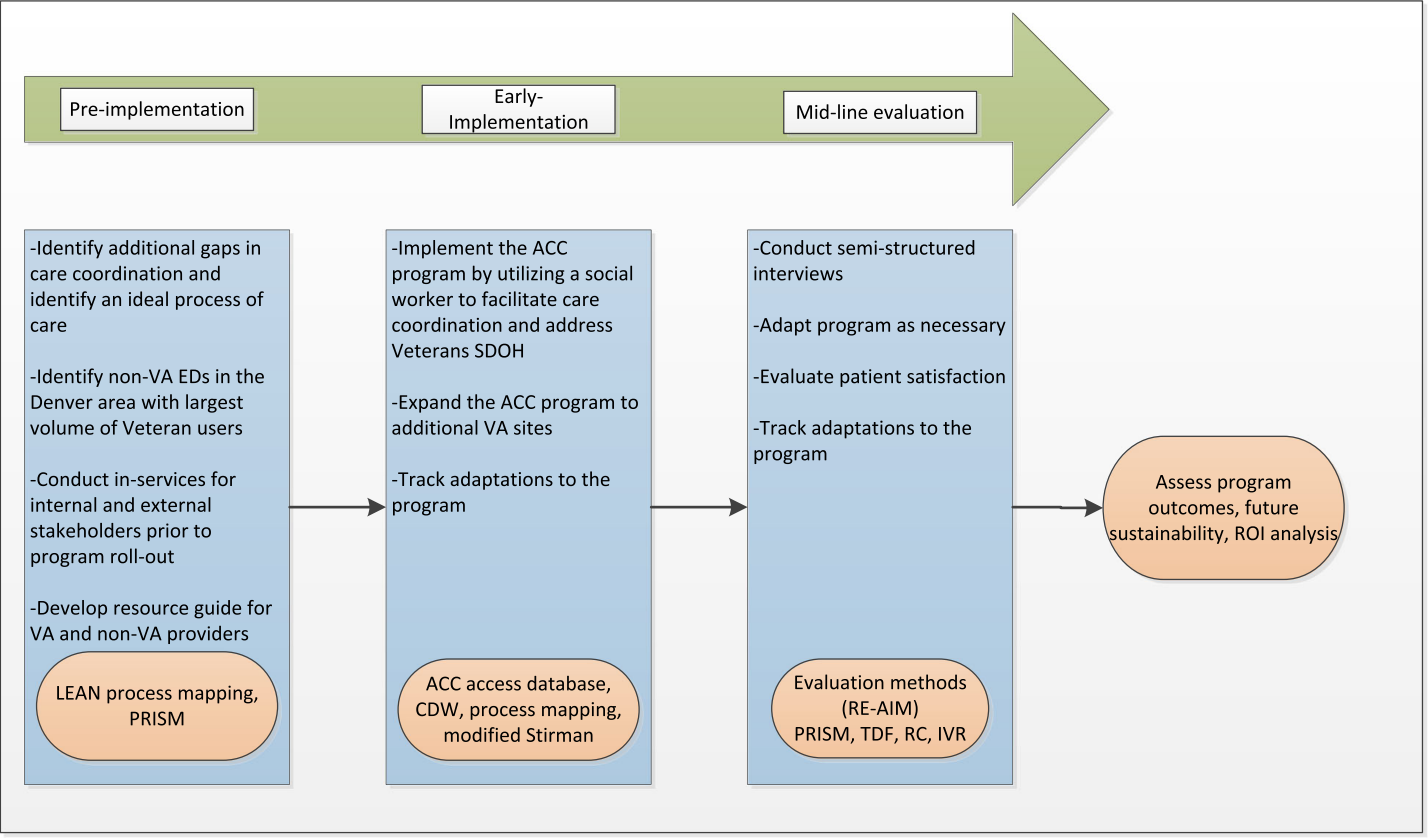
The Advanced Care Coordination Program Evaluation Process

We will evaluate our intervention and implementation strategies using a multi-level approach (Fig. 2 The Advanced Care Coordination Program Evaluation Process). Prior data from CHTP helped us identify gaps in care coordination and will inform the development of the ACC program during the pre-implementation stage. In addition to the data from CHTP, we will reach out to community partners and the VA to gain further insight into challenges in transitions of care. The pre-implementation assessment will be guided by the LEAN approach [31] and by the Practical, Robust Implementation and Sustainability Model (PRISM) framework [32]. Guided by this pre-implementation data we will tailor the ACC program to local settings and inform program rollout. We will use LEAN process mapping [31] and modified Stirman framework [33] to track program adaptations during early implementation and throughout the program implementation phases. We will conduct mid-line assessment to evaluate additional program adaptations. Moreover, data will be collected to assess effectiveness of Veteran and provider satisfaction with the program. The effectiveness of the program will be determined by utilization and stakeholder engagement, health outcomes of enrolled Veterans, as well as Veteran and provider satisfaction. The efficiency of the program will also be assessed by comparing the cost of implementation against observed health and economic outcomes. We will develop and deliver a toolkit of training materials to the VA OCC to facilitate wide dissemination of the comprehensive care coordination intervention.

We will primarily utilize the Reach, Effectiveness, Adoption, Implementation, and Maintenance (RE-AIM) [34] measures to determine intervention effectiveness as well as to evaluate the sustainability of the program.

Reach will be defined by the number, proportion, and representativeness of Veterans who received full and/or partial interventions through the ACC program. We will develop and utilize the ACC program access database to gather the number, proportion, and representativeness of all the Veterans referred to the ACC program. In addition, we will utilize the ACC program database to assess the number, proportion, and representativeness of Veterans who reached out to the ACC program after receiving our Care Card and the Care Card letter. Information on basic characteristics, including reason for non-VA ED visit, co-morbidities, level of acuity (assessed by the ACC social worker and documented in the ACC database as well as the Social Worker Comprehensive Assessment), and Care Assessment Need (CAN) scores of the Veterans who were enrolled into the ACC program will also be collected in the ACC program database. The above information will also be collected through the VA’s Computerized Patient Record System (CPRS) and Corporate Data Warehouse (CDW), fee basis data, and the stakeholder engagement spreadsheet developed for our program. These data will be obtained bi-annually from our program database. We will complete weekly database checks and will review the reports section in our database to assess the data.

Effectiveness will be evaluated in several ways. We will measure hospitalization and both VA and non-VA ED 90-day readmission rates for Veterans who received the ACC program intervention by reviewing our program database as well as CDW/fee basis data. This review will be conducted annually. We will obtain VA readmission data through CDW/fee basis data as well as through readmission rates in our program database. We have paid to obtain claims data from some of our partner non-VA hospitals and will use it to help measure 90-day readmission rates. Finally, we will determine the number, proportion, and representativeness of Veterans who received additional care and services due to the ACC program’s involvement.

We will analyze the information above by reviewing our program database and CDW/fee basis data annually. We will develop a survey to evaluate Veteran and provider satisfaction of care coordination and will utilize Interactive Voice Response (IVR) technology to implement the survey annually. We will evaluate the quality of transitions of care using the validated Care Transition Measure [35, 36]. In addition, we will measure patient satisfaction using validated questionnaires modified from the Survey of Healthcare Experiences in Patients [37]. We will also utilize the ACC program database as an evaluation tool to determine the number of referrals received from non-VA hospitals, the number of Veterans who received the ACC program intervention, and the number of Veterans who successfully completed the ACC program. We will utilize the database as a resource to determine program effectiveness and also use the data to evaluate Veteran adverse outcomes including 30-day hospital readmission rates and non-VA ED utilization rates, and death among those who have received interventions from the ACC program. We will use CDW data to compare these adverse outcomes for the ACC patients with those who did not participate in the ACC program but utilized non-VA EDs.

Adoption will be measured by the number, proportion, and representativeness of times that non-VA EDs notify the ACC program of a Veteran’s non-VA ED visit/discharge. We will collect information through the notification method checklist for each referral (i.e. phone call, fax, etc.) in our program database. We will also measure the number and roles of VA providers who collaborate with and provide referrals to the ACC program throughout the duration of the intervention. We will utilize CPRS notes as well as our program database to obtain this information. The evaluation team will have regular database checks and data will be pulled bi-annually for further analyses.

Implementation will be assessed by determining barriers and facilitators to implementation as well as through an economic evaluation that includes measuring costs associated with implementation and comparing them to potential cost offsets and health outcomes gained. We will track adaptations to the intervention and will complete a final assessment post-intervention completion to determine fidelity to the program delivery. The secure access database we create for the ACC program will be our primary measurement tool to track the ACC program interventions for each core component. The ACC social worker will enter data for the core components into the database in real-time. Database checks to see all or part of the core component completion will be done bi-annually. We will utilize the database to measure the acuity levels of the Veterans, the quantity of Veterans who completed all four core components, the length of time the Veterans are enrolled in the ACC program, the interventions Veterans have received from the ACC social worker, and readmission rates for Veterans who have been enrolled into the ACC program. Our health economist will complete a tool that tracks all implementation costs and will analyze it in the final assessment of the program. At the completion of the program we will execute a final analysis of implementation costs. We will complete qualitative interviews as a final assessment at the end of data collection to evaluate our implementation.

We will employ conventional content analysis study design to collect and analyze qualitative data for program implementation and evaluation. We will collect qualitative and process data during our mid-line evaluation 8 months after program rollout to inform the evaluation of the implementation outcomes. We will develop and then conduct semi-structured interviews guided by the PRISM framework [32] and the Theoretical Domains Framework (TDF) [38]. These interviews will be conducted during a mid-line evaluation 8 months after program rollout. They will provide us with feedback on the intervention implementation context, perceived barriers and facilitators to the ACC program implementation, and overall opinions and feedback on the program components, strategies, and program delivery, as well as suggestions for improvement. We will use convenience sampling initially, followed by both snowball and purposeful sampling. We will use snowball sampling for identification of VA and non-VA personnel involved in the care transition process. However, we will not interview all of the individuals, as some of their roles do not directly relate to our program implementation. We will aim to obtain as many different perspectives as possible and plan to conduct three interviews at each site, or as many as deemed feasible until saturation is reached. Interviews will be audio-recorded and transcribed verbatim.

Atlas.ti will be used to manage qualitative content. Transcripts will be organized and labeled by role and site in Atlas.ti to ensure ability to examine site-specific and role-specific information as well as overall data. We will use team-based consensus building to create a codebook and analyze for emerging themes. Analysis codes will be organized into domains and larger themes as they emerge. Key findings from all coded interviews will be summarized into one process map and process narrative. Variations in the process of transferring patients from non-VA EDs to the assigned VA primary care team will be included in the process narrative. A team of experienced qualitative analysts will conduct analyses using inductive and deductive approaches.

Our qualitative analysts will create a process map to depict the ideal transitions of care process. On a monthly basis, they will collaborate with the ACC social worker to revisit the program’s process and will update the process map to reflect changes and adaptations to the ACC program. Information to inform the process map will be collected throughout the implementation of the program. Any adaptations will also be recorded in our adaptations spreadsheet. This iterative process will allow the team to analyze, revise, and adapt our program until an ideal process is reached. We will train the ACC social worker at each site to use the Lean Six Sigma approach [31] to map his/her intervention process and to keep track of any process changes. During evaluation periods, an observer will view the ACC social worker process and update the process maps as needed. Process maps will be analyzed during the summative evaluation with a goal of informing implementation outcomes. A team of experienced qualitative analysts will conduct the analyses.

We will track adaptations to the program as its implementation at each site and changes that are made to make the program fit to each local context. Adaptations will be tracked by the ACC social worker on monthly basis. These adaptations will be documented through changes to process maps and in a modified Stirman spreadsheet. Additional data will be collected through semi-structured interviews that we will develop approximately 6 months after the rollout and at the end of outcome data collection. Adaptations data will be analyzed during the summative evaluation with a goal of informing implementation outcomes.

Maintenance will be measured by assessing local adaptability and continued implementation of the ACC program after the funding period ends through internal and external communication. We will also determine the maintenance of the ACC program through rapid prototyping and the feasibility of intervention expansion. We will measure this through our quantity of expansion sites and their ability to implement the program.

#### Other approaches

We will administer the Relational Coordination Survey, in partnership with RC Analytics [39, 40], to understand the state of relational coordination between non-VA and ECHCS providers and the ACC program staff. Relational coordination is a mutually reinforcing process of communication and relating for the purpose of task integration. The Relational Coordination Survey assesses the perceived frequency, timeliness, and accuracy of communication, the extent of problem-solving communication, shared knowledge and mutual respect between and among health care providers engaged in the ACC program. Targeted interventions will be implemented to address gaps in relational coordination to ensure quality teamwork and support healthy, learning organizations [39, 40].

## Discussion

The ACC program will promote care coordination for dual-use Veterans who access non-VA EDs by utilizing a social worker to provide longitudinal care coordination and resource referrals with an emphasis on SDOH. By expanding the work done with the CHTP, the ACC program will bridge additional identified gaps in care coordination for dual-use Veterans and provide smooth transitions of care back to their VA primary care.

This program may be constrained by a few limitations. The program will rely upon receiving notifications from non-VA ED staff when Veterans access their non-VA ED. It is not guaranteed that non-VA staff will incorporate this process into their workflow. However, based upon interviews with stakeholders from non-VA EDs, we expect non-VA EDs to embrace and collaborate with our program. Additionally, Veterans may be readmitted to non-VA EDs or to the VA ED while enrolled in this program, which could cause challenges for continuity of care. Limitations regarding sustainability may arise due to extra cost associated with an additional full-time equivalent (FTE) for a social worker. An economic evaluation will provide further information regarding efficiency for this program. We did not pay for claims data at each partner hospital, and will not have access to admission or utilization readmission rates data at these non-VA ED locations. This could limit the data we have access to when we are evaluating adverse outcomes through 90-day readmission rates. Finally, the ACC program will initially be implemented at two sites with the hope of expanding to more locations in the future. We will initially have a limited cohort; thus, this quality improvement program may not be generalizable to other sites or populations in the beginning phases of implementation.

Despite these limitations, the ACC program has numerous strengths. The ACC program is an innovative program within the ECHCS that directly partners with non-VA EDs to facilitate efficient use of health care resources and to reduce the utilization of non-VA EDs due to SDOH issues and to provide Veterans with enhanced continuity of care. The ACC program will implement system changes through offering direct phone and fax lines for the ACC social worker to enhance non-VA providers’ ability to collaborate with and coordinate care for Veterans with ECHCS providers, ultimately improving the relationship between the ECHCS and non-VA hospitals. Additionally, through regular in-services with non-VA providers, the ACC program will enhance community care provided to Veterans by educating non-VA providers about the process of accessing various services provided through the ECHCS. If successful, this program will enhance care provided to dual-use Veterans and will increase efficient use of resources.

The ACC program intervention is designed to provide support to Veterans during a vulnerable period of transition from hospital to home. The ACC program will offer case management services to Veterans during this time, linking them to appropriate resources and enhancing their stability in order to reduce potential non-VA and VA ED or hospital readmissions. The ACC program consists of a multidisciplinary team to effectively discuss complex cases, to review best ways to improve care for dual-use Veterans, and to ultimately link dual-use Veterans to comprehensive services with the intention of providing quality care tailored to each Veteran’s specific needs.

### Contribution to practice

The ACC program will improve care coordination for dual-use Veterans. By expanding the CHTP to incorporate longitudinal care coordination and SDOH, the ACC program will provide the necessary steps to close gaps in care coordination and to ensure quality transitions of care for Veterans who access non-VA EDs.

## Data Availability

The datasets generated and/or analyzed during the current study are not publicly available due to identifying nature of patients and providers. Furthermore, the VA claim data has patient data that is not to be shared publicly. However, how data was collected and managed can be shared including the CHTP interview guides via the corresponding author on reasonable request. This manuscript was formatted according to the SPIRIT guidelines (Chan A-W., Tetzlaff J., Gøtzsche P., Altman D., Mann H., Berlin J., et al. SPIRIT 2013 explanation and elaboration: guidance for protocols of clinical trials. BMJ. 2013;346:e7586.).

## Abbreviations

ACC: Advanced Care Coordination
CAN: Care Assessment Need
CDW: Corporate Data Warehouse
CHTP: Community Hospital Transitions Program
CPRS: Computerized Patient Record System
ECHCS: Eastern Colorado Health Care System
ED: Emergency Department
FTE: Full-Time Equivalent
IVR: Interactive Voice Response
ORH: Office of Rural Health
OVAC: Office of Veterans Access to Care
PACT: Patient Aligned Care Team
PRISM: Practical, Robust Implementation and Sustainability Model
QI: Quality Improvement
QUERI: The Quality Enhancement Research Initiative
RE-AIM: Reach, Effectiveness, Adoption, Implementation, Maintenance
RMR VAMC: Rocky Mountain Regional Veterans Affairs Medical Center
SDOH: Social Determinants of Health
TDF: Theoretical Domains Framework
TNP: Transitions Nurse Program
VA OCC: VA Office of Community Care
VA: Veterans Health Administration
VAMC: Veterans Affairs Medical Center
VISN: Veterans Integrated Service Network
